# Withdrawal

**DOI:** 10.1002/fsn3.3090

**Published:** 2022-10-19

Withdrawal: “Study of the influence of exogenous fibrolytic enzyme additive on chemical composition, fermentation characteristics, and nutritional value of brewer's spent grain,” by Khalil Abid, Jihene Jabri, Hela Yaich, Atef Malek, Jamel Rekhis, and Mohamed Kamoun, *Food Science & Nutrition*, 2022, 10, 1707–1713. https://doi.org/10.1002/fsn3.2743

The above article, published online in Volume 10, Issue 6 in Wiley Online Library (https://onlinelibrary.wiley.com/doi/full/10.1002/fsn3.2743), has been withdrawn by agreement between the authors, the Journal's Editor‐in‐Chief, Prof. Y. Martin Lo, and Wiley Periodicals, LLC. The withdrawal has been agreed because the article was published in error after having been rejected. The authors bear no fault, and we apologize for the error.

